# UvrY is required for the full virulence of *Aeromonas dhakensis*

**DOI:** 10.1080/21505594.2020.1768339

**Published:** 2020-05-20

**Authors:** Yi-Wei Chen, Wen-Hsuan Yeh, Hung-Jen Tang, Jenn-Wei Chen, Hung-Yu Shu, Yu-Chen Su, Sin-Tian Wang, Cheng-Ju Kuo, Yin-Ching Chuang, Chi-Chung Chen, Wen-Chien Ko, Chang-Shi Chen, Po-Lin Chen

**Affiliations:** a Department of Biochemistry and Molecular Biology, College of Medicine, National Cheng Kung University, Tainan, Taiwan;; b Institute of Basic Medical Sciences, College of Medicine, National Cheng Kung University, Tainan, Taiwan; c Division of Oral Biology and Medicine, School of Dentistry, University of California, Los Angeles, CA, USA; d Department of Medicine, Chi Mei Medical Center, Tainan, Taiwan; e Department of Microbiology and Immunology, College of Medicine, National Cheng Kung University, Tainan, Taiwan; f Department of Bioscience Technology, Chang Jung Christian University, Tainan, Taiwan; g Department of Medical Research, Chi Mei Medical Center, Tainan, Taiwan; h Department of Food Science, National Chiayi University, Chiayi, Taiwan; i Department of Internal Medicine, National Cheng Kung University Hospital, College of Medicine, National Cheng Kung University, Tainan, Taiwan

**Keywords:** *Aeromonas dhakensis*, two-component system, UvrY, pore-forming toxin, hemolysin, *Caenorhabditis elegans*

## Abstract

*Aeromonas dhakensis* is an emerging human pathogen which causes fast and severe infections worldwide. Under the gradual pressure of lacking useful antibiotics, finding a new strategy against *A. dhakensis* infection is urgent. To understand its pathogenesis, we created an *A. dhakensis* AAK1 mini-Tn10 transposon library to study the mechanism of *A. dhakensis* infection. By using a *Caenorhabditis elegans* model, we established a screening platform for the purpose of identifying attenuated mutants. The *uvrY* mutant, which conferred the most attenuated toxicity toward *C. elegans*, was identified. The *uvrY* mutant was also less virulent in C2C12 fibroblast and mice models, in line with *in vitro* results. To further elucidate the mechanism of UvrY in controlling the toxicity in *A. dhakensis*, we conducted a transcriptomic analysis. The RNAseq results showed that the expression of a unique hemolysin *ahh1* and other virulence factors were regulated by UvrY. Complementation of Ahh1, one of the most important virulence factors, rescued the pore-formation phenotype of *uvrY* mutant in *C. elegans*; however, complementation of *ahh1* endogenous promoter-driven *ahh1* could not produce Ahh1 and rescue the virulence in the *uvrY* mutant. These findings suggest that UvrY is required for the expression of Ahh1 in *A. dhakensis*. Taken together, our results suggested that UvrY controls several different virulence factors and is required for the full virulence of *A. dhakensis*. The two-component regulator UvrY therefore a potential therapeutic target which is worthy of further study.

## Introduction

*Aeromonas dhakensis*, also called *A. hydrophila* sub. *dhakensis*, is an endemic pathogen causes a variety of human diseases, including gastroenteritis, wound infections, septicemia, respiratory infections, hepatobiliary infections, and urinary tract infections worldwide [1–4]. The reported mortality rate among patients with *A. dhakensis* extra-intestinal infection varies from 25.5% to 37.5%, which is much higher than those infected by other *Aeromonas* species (ex. 0% to 14.0% in *A. veronii* infection) [5,6]. Previous studies have reported that *A. dhakensis* carries an array of virulence factors and exhibits the most potent toxicity to human blood cell lines among the tested *Aeromonas* species [6–9]. With the higher mortality and the abundance of virulence genes, *A. dhakensis* has become an extremely important pathogenic species. However, the whole picture of *A. dhakensis* pathogenesis remains unclear. Moreover, the regulation of virulence is not understood due to a lack of a comprehensive study.

UvrY has been known as the response regulator of the BarA-UvrY two-component system and is thought to function as a transcription factor in bacteria [10,11]. *A. dhakensis uvrY* has orthologs in many other species, such as *uvrY* in *E. coli, gacA* in *Pseudomonas, varA* in *Vibrio* and *sirA* in *Salmonella* [12–14]. Previous studies have reported that UvrY or its orthologues repress glycolysis and enhance gluconeogenesis with the aid of small RNAs *csrB* and *csrC* by inhibiting the translational initiator *csrA* [14,15]. Studies have also revealed that UvrY contributes to the activation of bacterial toxicity in many bacterial pathogens [12,13,16–18]. However, the route and regulon through which UvrY affects bacterial toxicity remain unclear.

In this study, we highlighted the contribution of UvrY to *A. dhakensis* infection and discovered that UvrY is responsible for the virulence through regulating an array of virulence factors in *A. dhakensis*. Of the virulence factors regulated by UvrY, pore-forming hemolysin Ahh1 contributes to the virulence of *A. dhakensis*. Taken together, our results suggested that the pathogenic role of UvrY is worth verifying in order to develop novel therapy against UvrY and UvrY-related virulent factors.

## Materials and methods

### Bacteria strains and culture conditions

All the bacteria strains and plasmids used in this study are listed in Table 2. *Aeromonas dhakensis* AAK1, *Staphylococcus aureus* subsp. *aureus* ATCC 29213, *Bacillus subtilis* 3610, and *Escherichia coli* strains OP50, DH5α SM10 λπ and S17-1 λπ were cultured in a Luria-Bertani (LB) broth at 37°C for 16 hours. *A. dhakensis* AAK1 deletion and transposon mutants were cultured with 100 μg/ml kanamycin (Kan), and complement strains were cultured with 50 μg/ml chloramphenicol (Cm) in LB broth at 37°C for 16 hours. *E. coli* SM10 λπ pBSL180 strain was cultured with 50 μg/ml Kan in LB broth at 37°C for 16 hours.

**Table 1.** Summary of the characteristics of 31 mutant strains.

bacteria		primary	secondary				tertiary		Strain/Gene description	
		Liquid assay score	Swimming (cm)	Swarming (cm)	Auxotroph	Growth defect	Biofilm (cm)	Plate assay median survival (day)		
	AAK1	0	3.10 ± 0.39	0.67 ± 0.06	X	X	1.70 ± 0.06	2		*Aeromonas dhakensis* AAK1
	ATCC 29213	–	0.73 ± 0.05	–	–	–	–	–		*Staphylococcus aureus* ATCC 29213
	3610	–	–	1.60 ± 0.10	–	–	–	–		*Bacillus subtilis*
	OP50	5	–	–	O	–	0.28 ± 0.08	–		*Escherichia coli* OP50
1	AA078G03	5	2.73 ± 0.05	0.67 ± 0.06	X	X	0.53 ± 0.12	5	*uvrY*	Response regulator UvrY
2	AA160E02	5	2.76 ± 0.15	0.63 ± 0.06	X	O	1.08 ± 0.17	2	*fbp*	Fructose-1,6-bisphosphatase
3	AA085D09	4	3.00 ± 0.09	0.57 ± 0.06	X	X	1.09 ± 0.11	2	*gltA*	Type ll citrate syntheses
4	AA092D12	3	1.06 ± 0.05	0.67 ± 0.12	X	X	0.62 ± 0.09	2	*aldH*	Acetaldehyde dehydrogenase
5	AA089H09	2	0.80 ± 0.10	0.67 ± 0.06	X	O	0.62 ± 0.08	2	*flhA*	Flagellar biosynthesis protein FlhA
6	AA089H12	2	0.63 ± 0.05	0.60 ± 0.10	X	X	0.63 ± 0.06	3	*flgF*	Flagellar basal-body rod protein
7	AA092D09	2	1.00 ± 0.10	0.70 ± 0.10	X	X	0.65 ± 0.08	2	*fliK*	Putative flagellar hook-length control protein
8	AA111H04	2	3.03 ± 0.15	0.63 ± 0.06	O	X	1.10 ± 0.14	2	*panC*	Pantoate–beta-alanine ligase
9	AA119A04	2	2.73 ± 0.15	0.67 ± 0.15	X	X	1.15 ± 0.11	2	*napA*	Periplasmic nitrate reductase, large subunit
10	AA130H03	2	0.56 ± 0.05	0.63 ± 0.06	X	X	0.62 ± 0.09	2	*fliF*	Flagellar M-ring protein
11	AA140E01	2	0.76 ± 0.05	0.67 ± 0.06	X	X	0.63 ± 0.08	2	*fliH*	Flagellar assembly protein
12	AA225A11	2	0.70 ± 1.35	0.63 ± 0.06	X	X	0.60 ± 0.11	3	*flgI*	Flagellar P-ring protein
13	AA255H03	2	0.63 ± 0.05	0.63 ± 0.15	X	X	0.64 ± 0.06	3	*argD*	Acetylornithine aminotransferase
14	AA072G07	1	2.83 ± 0.20	0.67 ± 0.06	O	O	0.46 ± 0.07	3	*flmH*	flagellar modification protein
15	AA076F05	1	2.63 ± 0.15	0.67 ± 0.06	X	O	0.16 ± 0.09	3	*flmD*	flagellar modification protein
16	AA084D04	1	1.00 ± 0.20	0.63 ± 0.06	X	O	0.45 ± 0.09	2	*fliG*	flagellar motor switch protein
17	AA087C12	1	0.63 ± 0.05	0.63 ± 0.12	O	O	0.15 ± 0.15	3	*pepQ*	proline dipeptidase
18	AA095G04	1	0.60 ± 0.10	0.60 ± 0.10	X	X	0.23 ± 0.12	3	*pepQ*	proline dipeptidase
19	AA113G01	1	0.83 ± 0.05	0.67 ± 0.06	X	X	0.14 ± 0.10	3	*motX*	Flagellar protein MotX
20	AA130G02	1	0.63 ± 0.05	0.67 ± 0.12	X	X	0.25 ± 0.11	3	*pepQ*	proline dipeptidase
21	AA135D01	1	2.73 ± 0.15	0.63 ± 0.12	X	X	0.26 ± 0.11	3	*napA*	nitrate reductase large subunit
22	AA152B02	1	1.46 ± 0.15	0.63 ± 0.06	X	O	1.03 ± 0.12	2	*cheV*	chemotaxis protein CheV
23	AA153A03	1	0.76 ± 0.05	0.60 ± 0.10	X	X	0.87 ± 0.10	3	*pepN*	aminopeptidase N
24	AA163E02	1	0.70 ± 0.10	0.60 ± 0.10	O	X	1.27 ± 0.17	5	*gabT*	4-aminobutyrate aminotransferase
25	AA164H03	1	2.63 ± 0.15	0.60 ± 0.10	X	X	1.46 ± 0.06	3	*ttrA*	tetrathionate reductase subunit A
26	AA166A11	1	1.43 ± 0.20	0.63 ± 0.12	X	X	1.35 ± 0.12	3	*cheW*	chemotaxis protein CheW
27	AA221A11	1	1.00 ± 0.10	0.67 ± 0.12	X	X	1.15 ± 0.09	2	*fliR*	flagellar biosynthesis protein
28	AA240A07	1	5.33 ± 0.51	0.63 ± 0.06	X	O	1.63 ± 0.23	5	*argH*	argininosuccinate lyase
29	AA262E01	1	2.03 ± 0.15	0.67 ± 0.06	X	O	1.06 ± 0.11	3	*flhF*	flagella biosynthesis protein FlhF
30	AA266A08	1	3.96 ± 0.15	0.67 ± 0.15	X	O	1.63 ± 0.10	3	*rsmB*	16 S rRNA methyltransferase
31	AA266A09	1	4.03 ± 0.15	0.67 ± 0.06	X	O	1.63 ± 0.11	3	*adhE*	acetaldehyde dehydrogenase

**Table 2.** Bacteria strains and plasmids used in this study.

Species	Host/Strain	Plasmid/Vector	Note	Reference
*A. dhakensis*	AAK1		*A. dhakensis* wild type	[32]
*A. dhakensis*	AA078G03		*uvrY* mini-Tn10 transposon mutant	This study
*A. dhakensis*	YQ327		*uvrY* deletion mutant; KanR	This study
*A. dhakensis*	YQ461	pwf417	*uvrY* complement of *uvrY* deletion mutant; KanR, CmR	This study
*A. dhakensis*	YQ462		*barA* deletion mutant; KanR	This study
*A. dhakensis*	YQ463	pwf417	*uvrY* complement of *barA* deletion mutant; KanR, CmR	This study
*A. dhakensis*	YQ464	pwf418	UvrY(D54A) complement of *uvrY* deletion mutant; KanR, CmR	This study
*A. dhakensis*	YQ465	pwf419	UvrY(D54E) complement of *uvrY* deletion mutant; KanR, CmR	This study
*A. dhakensis*	YQ466		*ahh1* deletion mutant; KanR	This study
*A. dhakensis*	YQ467		*aeroA* deletion mutant; KanR	This study
*A. dhakensis*	YQ468		*collagenase* deletion mutant; KanR	This study
*A. dhakensis*	YQ469	pwf420	*ahh1* complement of *ahh1* deletion mutant; KanR, CmR	This study
*A. dhakensis*	YQ470	pwf420	*ahh1(pahh1)* complement of *uvrY* deletion mutant; KanR, CmR	This study
*A. dhakensis*	YQ471	pwf421	*ahh1(pgyrB)* complement of *uvrY* deletion mutant; KanR, CmR	This study
*E. coli*	OP50		normal food source of *C. elegans*; Uracil auxotroph; Biofilm deficiency	[23]
*E. coli*	SM10 λπ	pBSL180	conjugative plasmid pBSL180 with mini-Tn10 transposon; KanR	[38]
*E. coli*	S17-1 λπ	pwf300	conjugative plasmid for generating deletion mutant; modified from pBSL180; *sacB*^+^, IS10^−^; KanR	This study
*E. coli*	S17-1 λπ	pwf412	conjugative plasmid for generating *uvrY* deletion mutant; KanR	This study
*E. coli*	S17-1 λπ	pwf413	conjugative plasmid for generating *barA* deletion mutant; KanR	This study
*E. coli*	S17-1 λπ	pwf414	conjugative plasmid for generating *ahh1* deletion mutant; KanR	This study
*E. coli*	S17-1 λπ	pwf415	conjugative plasmid for generating *aeroA* deletion mutant; KanR	This study
*E. coli*	S17-1 λπ	pwf416	conjugative plasmid for generating *collagenase* deletion mutant; KanR	This study
*E. coli*	S17-1 λπ	pBBRMCS1	conjugative broad host plasmid for generating complement strain; CmR	[29]
*E. coli*	S17-1 λπ	pwf417	modified pBBRMCS1 for generating *uvrY* complement strain; CmR	This study
*E. coli*	S17-1 λπ	pwf418	modified pBBRMCS1 for generating UvrY(D54A) complement strain; CmR	This study
*E. coli*	S17-1 λπ	pwf419	modified pBBRMCS1 for generating UvrY(D54E) complement strain; CmR	This study
*E. coli*	S17-1 λπ	pwf420	modified pBBRMCS1 for generating *ahh1(pahh1)* complement strain; CmR	This study
*E. coli*	S17-1 λπ	pwf421	modified pBBRMCS1 for generating *ahh1(pgyrB)* complement strain; CmR	This study
*E. coli*	DH5α		A nontoxic control in *C. elegans* survival assay	[62]
*E. coli*	DH5α	pwf421	*ahh1(pgyrB)* complement strain; CmR	This study
**S. aureus**	ATCC 29213		swimming negative control	[63]
*B. subtilis*	3610		swarming positive control	[22]

### Worm strains and culture conditions

The *C. elegans* wild-type Bristol N2 strain and PD4793 *mIs10*[*myo-2p*::GFP + *pes-10p*::GFP + gut-promoter::GFP] worms were provided by the Caenorhabditis Genetics Center (CGC), which is supported by the National Institutes of Health – Office of Research Infrastructure Programs (P40 OD010440). The animals were maintained on Nematode Growth Medium (NGM) with *E. coli* strain OP50 as the normal food source.

### A. dhakensis mini-Tn10 transposon library

The library was built through conjugation with pBSL180, which contains a mini-Tn10 transposon and kanamycin resistant gene *nptII*. Overall, 500 μl of *A. dhakensis* AAK1 and *E. coli* SM10 λπ pBSL180 were cultured overnight and mixed gently. After centrifuging at 12,000 x *g* for 3 minutes, the supernatant was removed, and the bacteria pellet was washed 3 times with 1 ml of a conjugation buffer (10 mM MgSO_4_). The washed bacteria mixture was spread evenly on a 0.45 μm membrane filter. The filter was incubated continuously at 37°C on an LB plate for 5 hours, and then on an LB plate with 1 mM IPTG for 1 hour, and finally on an LB plate for 1 hour. Bacteria were collected from the filter through washing with 10 ml deionized sterile water and 50 μl of the bacteria were cultivated on Aeromonas Agar (LAB167, LAB M^TM^) with 100 μg/ml Kan at 37°C for 16 to 18 hours. Generally, there were 150 colonies growing on each plate. Each colony was picked and cultured in a well with 100 μl LB and 100 μg/ml Kan in a 96-well plate (353,072, Falcon) at 37°C for 16 to 18 hours. The 96-well plates were stored at −80°C after adding 100 μl LB with 30% glycerol to each well and mixing carefully.

### Liquid toxicity (LT) screen

By using 96 solid pin multi-blot replicators, each 96-well plate in the transposon library was triplicated to another 96-well plate with 180 μl LB in each well and kept at 37°C for 16 to 18 hours. After centrifuging at 4,000 x *g* for 15 minutes, the supernatant was removed, and the bacteria pellet was resuspended with 200 μl S medium combined with approximately 20 to 30 synchronized PD4793 larva 4 (L4) stage worms. The plates were incubated at 25°C for 60 hours with constant shaking at 80 x *g*. The survival rate and GFP expression rate of the worms in each well were measured.

### Arbitrary PCR

The arbitrary primed PCR was used to identify the insertion site of the mini-Tn10 transposon of *A. dhakensis* transposon mutants [19,20]. Briefly, a single colony PCR with an arbitrary primer (Arb1 or Arb6) and a transposon primer (nptR) were used for a first-round PCR. The PCR program was set as follows: 95°C for 10 min, followed by six cycles at 95 ^o^C for 30 s, 30 ^o^C for 30 s, and 72 ^o^C for 2 min; 30 cycles at 95 ^o^C for 30 s, 45 ^o^C for 30 s, and 72 ^o^C for 2 min, and finally, at 72 ^o^C for 3 min. One-tenth of the cleaned-up PCR product was then used as the DNA template for a second-round PCR with the Arb2 primer and a nested transposon primer (nptR1). The PCR program was set as follows: 95 ^o^C for 1 min, followed by 30 cycles at 95 ^o^C for 30 s, 55 ^o^C for 30 s, and 72 ^o^C for 2.5 min, and finally, then 72 ^o^C for an additional 4 min. The second-round PCR products were electrophoresed, and bands over 1,000 bp were gel-extracted and sequenced using Arb2 and nptR1 primers. The primers mentioned above are listed as follows:

Arb1: GGCCACGCGTCGACTAGTACNNNNNNNNNNGATAT

Arb6: GGCCACGCGTCGACTAGTACNNNNNNNNNNACGCC

Arb2: GGCCACGCGTCGACTAGTAC

nptR: GCATTGCATCAGCCATGATGG

nptR1: CATCAGAGCAGCCGATTGTCTG

### Swimming and swarming motility

LB with 0.3% Bacto agar (Difco Laboratories) was used to determine swimming motility, and LB with 0.5% Bacto agar was used to measure the swarming motility of all mutants [21,22]. The plates were incubated at 37°C overnight, and motility was measured by examining the distance bacteria migrated from the center toward the periphery of the plate.

### Auxotroph test

M9 minimal media was used to incubate all mutants for the auxotroph test [23]. Briefly, a colony of mutants was picked into at least 6 wells of a 96-well plate. Each well contained 200 μL M9 media. The plate was incubated at 37°C for 18 hours. The bacterial growth, as determined by the OD600 values of each well, was recorded.

### Growth analysis of bacteria

The OD-Monitor C&T (Taitec, Japan) was used to continuously observe the growth of bacteria. Briefly, of each mutant colony was seeded into 5 ml LB broth and incubated at 37°C in the OD-Monitor. The growth of bacteria measured according to the OD600 value was detected every 30 minutes.

### Biofilm formation assay

Crystal violet staining was used to detect the expression of biofilm [24]. Briefly, a colony of mutants was picked into at least 6 wells of a 96-well plate. Each well contained 200 μl of LB broth. The plate was incubated at 37°C for 18 hours. After removing the culture media and washing three times with deionized water, 125 μl of a 0.1% crystal violet solution in water was added to each well and left for 15 min at room temperature. After washing three times with deionized water, 125 μl of 30% acetic acid in water was added to each well to solubilize the crystal violet dye. The OD600 values of each well were recorded.

### C. elegans survival assay

The procedures followed those of previous studies [25–27]. Synchronized L1 worms were seeded onto an ENG plate with *E. coli* OP50 and incubated at 20°C for 44 hours. Approximately 50 L4 worms were transferred onto an NG plate spread and cultured with OD600 = 2.0 *A. dhakensis* 30 μl. Live worms were counted and transferred daily. All the survival assays were conducted at on 20°C.

### Deletion mutant of a. dhakensis

To generate deletion mutations, the host cell proteins driving homologous recombination between the allelic exchange vector and the recipient chromosome were used as the deletion mutants of *A. dhakensis* AAK1 constructed via allelic exchange, using the vector pwf300 containing a suicide gene *sacB* (levansucrase) and a kanamycin resistant gene *nptII* as selective markers [28]. Briefly, about 500 bp of the 3ʹ and the 5ʹ regions of target gene were cloned into the left and right site of *nptII*. The plasmid was electroporated into *E. coli* S17-1 λπ and transferred into AAK1 through conjugation. The transconjugants that grew on Aeromonas Agar (LAB167, LAB M^TM^) was then cultured on an LB plate with 6% sucrose and 50 μg/ml Kan at 20°C to allow the levansucrase convert sucrose to form levans and force the consummation of homologous recombination.

### Generation of complement strain

Broad host plasmid pBBRMCS1 was used to create the complement strain [29]. In brief, the target gene was cloned into the multiple cloning sites of pBBRMCS1, and the reconstructed plasmid was transferred into *E. coli* S17-1 λπ. The conjugants were spread on Aeromonas Agar (LAB167, LAB M^TM^) plates containing 50 μg/ml Cm and 100 μg/ml Kan.

### Cell cytotoxicity

The C2C12 mouse fibroblast cells were cultured in a complete medium consisting of Dulbecco’s Modified Eagle’s medium (DMEM, Gibco) and 10% fetal bovine serum (FBS, Invitrogen) at 37°C with 5% CO_2_ [6]. The C2C12 fibroblast cells were seeded into 24-well plates (5x10^4^ cells/well) and incubated overnight. With a MOI of 20 ratio, refreshed *A. dhakensis* were added to DMEM without FBS. After 2 hours of infection, the culture medium was examined for the release of lactate dehydrogenase (LDH) using a CytoTox 96 kit (Promega). A 0.1% Triton X-100 solution was used as a positive control, and serum-free Roswell Park Memorial Institute (RPMI) medium was used as a negative control. The cytotoxicity activity was expressed as the mean of triplicate measurements of released LDH levels compared with Triton X-100 exposure (defined as 100% cytotoxicity).

### Mice survival

Based on our previous study [6], 6–10 week-old female BALB/c mice weighing 18–22 grams were obtained from the National Laboratory Animal Center. Each mouse was injected intraperitoneally with 100 μl containing various bacterial loads of *A. dhakensis*. Three mice were tested for each experimental group in each experiment. The mice survival was monitored daily for 3 days. All the animal experiments in this study were carried out in strict accordance with the recommendations set forth in the guidelines for the Committee of Laboratory Care and Use of Animals, developed by National Cheng Kung University. The protocol was ethically approved by the Institutional Animal Care and Use Committees and National Cheng Kung University (Permit No: 107,080).

### Serum resistance

The susceptibility of the bacterial isolates to human serum was analyzed as described previously [30]. In brief, twenty-five microliters of the bacterial suspension (about 2 × 10^6^ CFU) were mixed with 75 μl of pooled normal human serum in microtiter plates and then incubated at 37 °C for 3 hours. The test was performed in triplicate and the number of recovered bacteria was determined.

### Determination of in vivo bacterial load

Mouse organs were individually homogenized in a suitable volume of PBS using the Micro Tissue Homogenizer (Pierce Chemical Co., Ill., USA). Homogenates were serially diluted in PBS and plated on TSB to determine bacterial numbers. The detection limit was defined as 30 to 300 CFU, as a modification of a previous report [31].

### Blood cytokines

Interleukin 1β (IL-1β), monocyte chemoattractant protein-1 (MCP-1/CCL2) and tumor necrosis factor α (TNF-α) were measured using ELISA (R&D Systems, Minneapolis, MN) [31].

### RNAseq

Approximately 5 mL of LB broth containing bacteria grown overnight were collected for RNA extraction. RNAs for sequencing were prepared using the Ion Total RNA-Seq Kit v2 (Ion Torrent™) according to the manufacturer’s instructions. Quality and base trimming were performed on Ion OneTouch™ 2 System reads from RNA libraries. The sequencing raw data was mapped according to the reference genome [32]. The normalized count of sequencing reads (RPKM) was determined for each gene. The raw data and normalized results were updated to GEO (accession number: GSE130707).

### qRT-PCR

The expression of the UvrY-regulated genes and *gyrB* (internal control) was measured using quantitative real time-PCR (qRT-PCR,) as described previously [26,33]. Approximately 3 ml of LB broth containing bacteria that had been cultivated overnight were collected for RNA extraction. RNA samples (2 μg for each experimental group) were converted to cDNA via reverse transcription. All qRT-PCRs were carried out using the FastStart Universal SYBR Green Master kit (ROX) according to the manufacturer’s specifications and analyzed on a StepOnePlus Real-Time PCR System. The expression level of each target was collected and compared to the internal control and conduct as a ΔCt value,where the Ct was equal to the number of PCR cycles required to amplify a given gene from a cDNA population. The fold-change values were estimated using the following equation: Fold change = 2^[-ΔCt (AAK1)]^/2^[-ΔCt (OP50)]^. The qRT-PCR primers are listed below:

Zn-dependent exopeptidase M28 Forward primer: TACAGCTTCGATCCCAAGGTA

Zn-dependent exopeptidase M28 Reverse primer: CCCTCAGGGTAGCCATCATA

pilus *pilN* Forward primer: TGTCAAATATAAACCTCCTTCCCT

pilus *pilN* Reverse primer: CCGTGATGGCCATGAAGATA

pilus *pilO* Forward primer: CAAGGATCGTCATTCTGGAGTC

pilus *pilO* Reverse primer: ACCGTCTTGCCGTTGTATTTA

pilus *pilQ* Forward primer: CTGCAGGAGGTCAAAGTCAA

pilus *pilQ* Reverse primer: TAGGAGAGCCGATCGGTAAA

exonuclease Forward primer: GTACCGACACCTACTCCTACA

nuclease Reverse primer: AATCCTCGATGCCGATCAC

exotoxin *exoA* Forward primer: TCATCAGGTTTGCGCCATA

exotoxin *exoA* Reverse primer: GATGAGATTGTGGACCGGATAG

hemolysin *ahh1* Forward primer: GATCTCGCGATGCTCATACTT

hemolysin *ahh1* Reverse primer: AGGATTACCGCTTCAGCATC

protease *lasA* Forward primer: CACTTCTCCCTGCTCTACAAC

protease *lasA* Reverse primer: TTGCAGTTGTCGTCGTAGTT

chitinase *chiA* Forward primer: GGTGAACTTGCGACCATAGA

chitinase *chiA* Reverse primer: CCGCTCAAGGAGAACAACA

collagenase Forward primer: GAAGAGCAGCAGTTCCATCA

collagenase Reverse primer: GGAGGAGTCGAAGATTACCAAC

lipase *estA* Forward primer: GACCGGCAGCTTCTTCAA

lipase *estA* Reverse primer: CAGATGGTTCATCCGGTAGTC

lipase chaperone *chaP* Forward primer: CTGCAGGCTCGCTATGA

lipase chaperone *chaP* Reverse primer: CTCGTCGAACAGCACCT

aerolysin *aeroA* Forward primer: GTATCCCAAATAGTGGGCAAGA

aerolysin *aeroA* Reverse primer: ATATTCCGGCAGGTGATGAAG

gyrase subunit B *gyrB* Forward primer: CCTGCTGCTAACCTTCTTCTATC

gyrase subunit B *gyrB* Reverse primer: CTTGCCCTTCTTCACCTTGTA

### RAST analysis

The RNAseq results were re-mapping using RAST software available online [34,35]. The virulence genes with expression levels indicating a 10-fold decrease in the *uvrY* mutant compared to wild type AAK1 and *uvrY* complement strains were analyzed.

### Pore-formation assay

Based on our previous studies [36,37], L4 stage N2 worms were infected with either *A. dhakensis* or Ahh1-overexpressed *E. coli*. At each time point, 10 worms were transferred to microcentrifuge tubes with M9 medium mixed with 5 mg/ml serotonin and 6.7 mg/ml propidium iodide dye and incubated for 30 min at room temperature while undergoing shaking. The worms were mounted on slides after being washed twice with M9 medium. The experimental animals were scored positive for pore-formation if at least one of the intestinal cells was stained with propidium iodide. Three independent repeats were performed in each treatment.

### Western blot

An analysis of Ahh1 hemolysin production of either *A. dhakensis* or *E. coli* was performed using a western blot method as described previously with some modifications [26]. In brief, 3 ml of LB broth containing bacteria cultivated overnight was collected to extract total protein using Bullet Blender® homogenization and precipitating with acetone. Rabbit polyclonal antibody to Ahh1 (Leadgene Biomedical Inc., Tainan, Taiwan) and rabbit monoclonal antibody to RpoB (EPR18704, abcam) were used for detection.

### Image

The worms were mounted on a 2% agarose gel with M9 medium on a slide. Two microliters of 1% sodium azide was added to paralyze the worms. The samples were covered with a slip and observed using a DIC or RFP filter from a fluorescent microscope (Nikon Eclipase Ti inverted microscope system) equipped with a CCD Camera (QImaging Retiga-2000 R Fast 1394).

### Statistical analysis

All experiments were performed a minimum of three times independently. The survival assay was analyzed using the Kaplan-Meier method, and survival difference was accessed using a log-rank test. The Student’s t-test was used to analyze the statistical results of the two different groups. A one-way ANOVA test was used to analyze the statistical values among the groups for one independent variable. Statistical significance was at P < 0.05. (***: P < 0.001; **: P < 0.01; *: P < 0.05) (Error bars: standard deviation, SD)

## Results

**A forward genetic screen to identify the *A. dhakensis* gene involved in the attenuation of *A. dhakensis* pathogenesis.**

**Figure 1.**
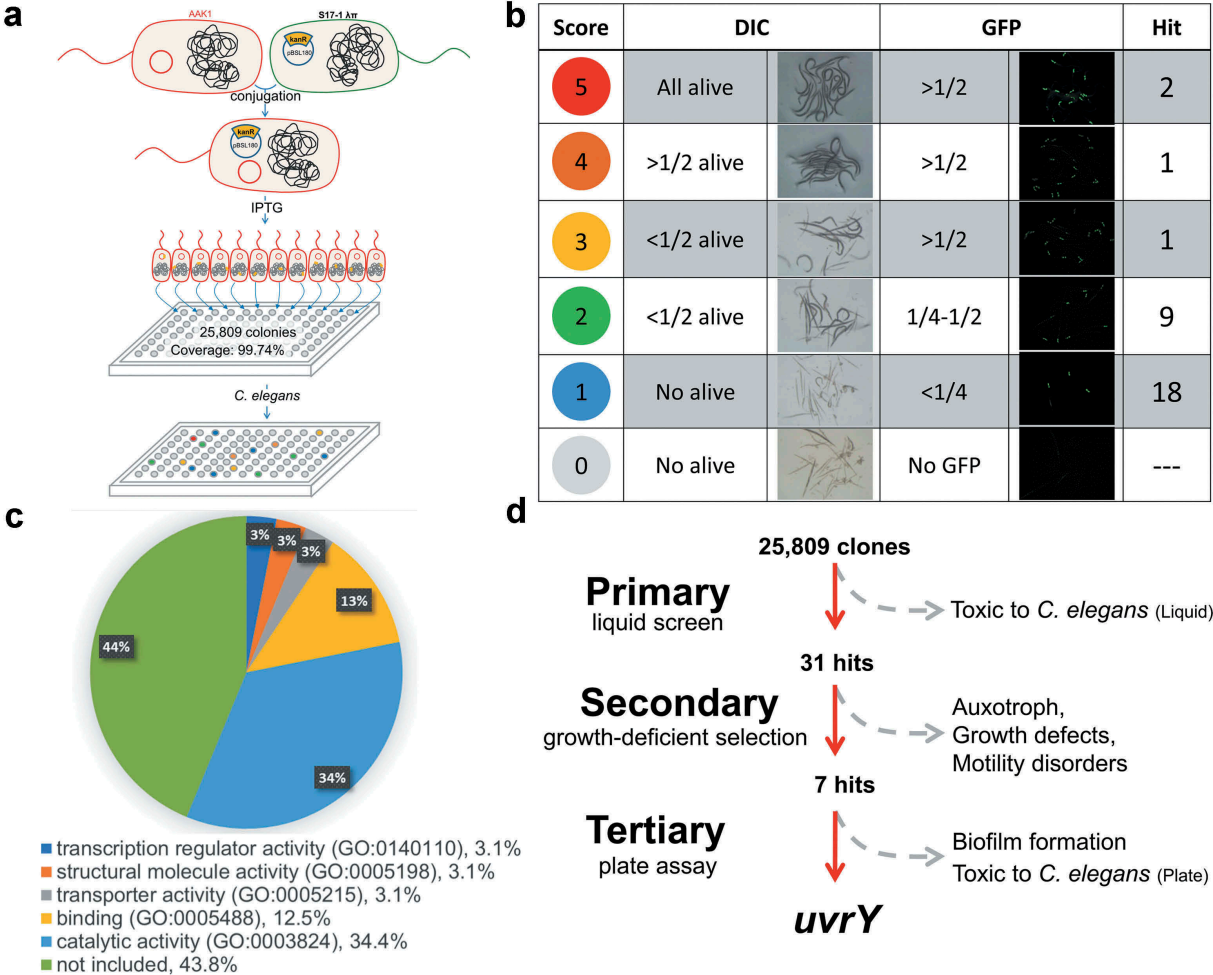
**Identification of genes participating in the virulence of *A. dhakensis***. (a) Diagram of the establishment of the *A. dhakensis* AAK1 mini-Tn10 transposon library. (b) Screen of transposon mutants with attenuated virulence. (c) GO analysis of candidate genes based on molecular function. (d) Identification of *uvrY* as a target gene. (***P < 0.001, **P < 0.01 and *P < 0.05).

We created a mini-Tn10 transposon library of *Aeromonas dhakensis* AAK1 [32] through conjugation with plasmid pBSL180 in *E. coli* SM10 (Figure 1(a)) [38]. Our transposon library contained 25,809 clones which gave a 99.74% coverage [39] of *A. dhakensis* AAK1 genome. By using bacteriovorus nematode *Caenorhabditis elegans* as the model host, we screened the transposon library to isolate mutants with attenuated virulence. The screen scores were measured by the survival of worms and the retained green fluorescence among PD4793 worms after infection in liquid toxicity assay [25,27]. Thirty-one attenuated mutants were selected for further analysis (Figure 1(b)). The transposon-inserted genes responsible for the 31 mutants are summarized in Table 1. By the Gene Ontology (GO) analysis [40], the mutated genes were categorized based on their previously known function (Figure 1c and S1 Fig). According to the description of each hit on the PANTHER website, most of them were reported to involve in metabolism and flagellar biosynthesis.

To exclude the genes responsible for mobility, growth and development, phenotypes of the swimming (S2A Fig) [21], swarming (S2B Fig) [21,22], auxotroph (S2C Fig) [41], and growth deficiency (S3 Fig) of these 31 mutants were tested. The biofilm formation ability (S2D Fig) as well as virulence to *C. elegans* (S4 Fig) were also examined [26,27]. Following the selection protocol, we turned our attention to the two-component system mutant strain *uvrY* strain for further study (Figure 1(d)).

**The attenuated virulence of the *uvrY* mutant was not related to defects in bacterial physiological features**

**Figure 2.**
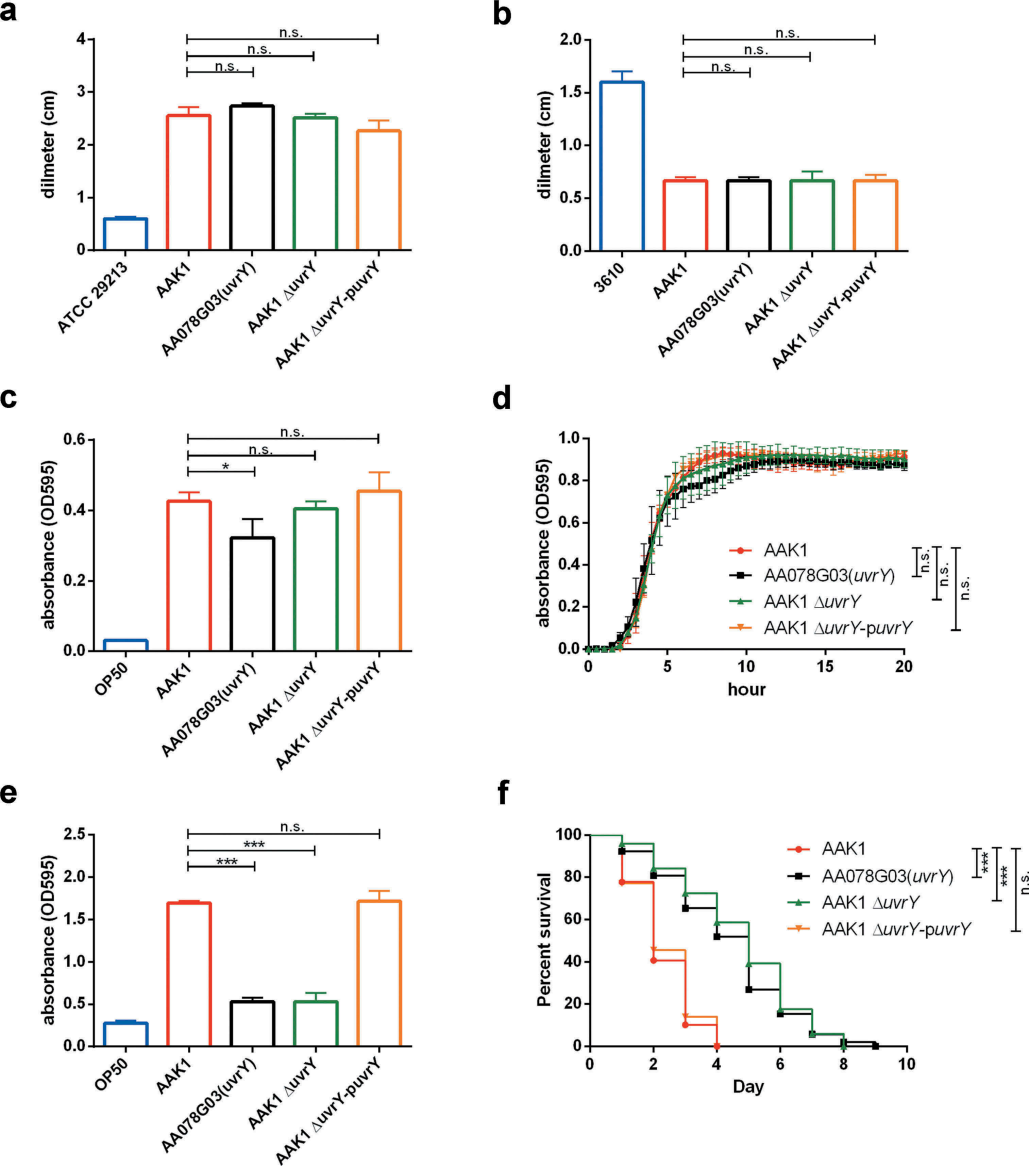
**UvrY dominates the virulence without altering the physiological functions of *Aeromonas dhakensis* in terms of** (a) swimming, (b) swarming, (c) auxotroph, (d) growth curve, (e) biofilm formation, and (f) virulence to *C.elegans. s. aureus* ATCC 29213 is a negative control strain for swimming. *B. subtilis* 3610 is a positive control strain for swarming. *E. coli* OP50 is a negative control strain for auxotroph, growth curve and biofilm formation. (***P < 0.001, **P < 0.01 and *P < 0.05).

To validate the virulence reduction of the *uvrY* transposon mutant, we generated an isogenic *uvrY* mutant of *A. dhakensis* AAK1 (S5 Fig) and rechecked the characteristics tested as discussed above. Compared to the *uvrY* transposon mutant, the motility (Figure 2(a,b)), auxotroph (Figure 2(c)), growth (Figure 2(d)), biofilm formation (Figure 2(e)), and toxicity to *C. elegans* (Figure 2(f)) remained unchanged in the *uvrY* isogenic mutant. The attenuation of virulence also displayed in the *uvrY* deletion mutant both *in vitro* (Figure 2(e)) and *in vivo* (Figure 2(f)). The biofilm formation level and toxicity to *C. elegans* of the *uvrY* complement strain (Δ*uvrY*-p*uvrY*) were both rescued (Figure 2(e-f)). Taken together, these findings suggested that *uvrY* contributes to the toxicity of *A. dhakensis* without altering its physiological features.

### *Two-component system barA-uvrY participates in the regulation of* a. dhakensis *toxicity.*

**Figure 3.**
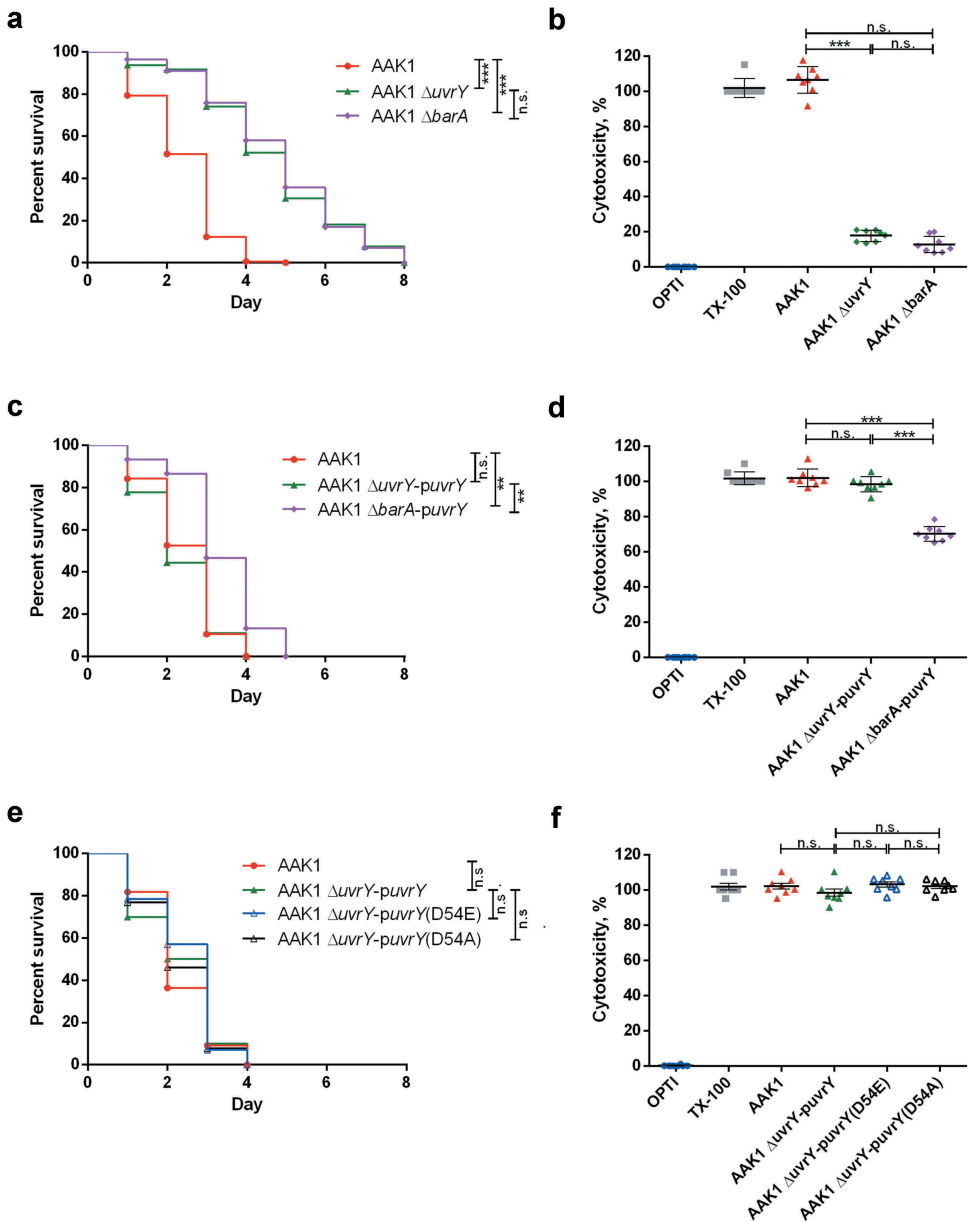
**The BarA-UvrY two-component system controls the toxicity of** **A. dhakensis AAK1**. the virulence of the barA mutant is similar to the *u*uvrY mutant in (a) *C. elegans* and (b) the C2C12 mouse myoblast cell line. complementation of uvrY rescues the toxicity not only in the *uvry* mutant but also in the *bara* mutant in (c) *C. elegans* and (d) the C2C12 mouse myoblast cell line. complementation of both UvrY D54E (constitutively active form) and UvrY D54A (inactive form) reverse the toxicity in (e) *C. elegans* and (f) the C2C12 mouse myoblast cell line. (***p < 0.001, **p < 0.01 and *p < 0.05).

Previous study of other bacteria has shown that UvrY is a response regulator that is phosphorylated by BarA [42]. To understand the role of BarA and the BarA-UvrY two-component system in the regulation of *A. dhakensis* toxicity, the correlation between UvrY and BarA was analyzed. In the *C. elegans* infection model, the virulence of the *A. dhakensis barA* isogenic mutant was attenuated as the *uvrY* mutant (Figure 3(a)). However, the toxicity of the *uvrY* complement in the *barA* mutant (Δ*barA-*p*uvrY*) was significantly increased (Figure 3(c)). A similar phenomenon was also observed in a cell infection model with C2C12 mouse myoblast (Figure 3(b-d)). These results suggested that the response regulator UvrY regulates the toxicity of *A. dhakensis* even without BarA.

Previous studies have found that the UvrY is phosphorylated by BarA at D54 residue [11,42,43]. To determine the importance of D54 residue of UvrY, we generated D54E (constitutively active form) and D54A (inactive form) mutants of UvrY. Interestingly, in both the *C. elegans* (Figure 3(e)) and C2C12 cell models (Figure 3(f)), mutation of D54 residue does not alter the toxicity of UvrY in *A. dhakensis*. These results suggested that BarA is not the only phosphate donor to UvrY, which is similar to previous findings in *E. coli* [44,45], and the phosphorylation of the D54 residue of UvrY is not essential for the virulence of *A. dhakensis*.

### *UvrY regulates the virulence of* a. dhakensis *in a mouse infection model*

**Figure 4.**
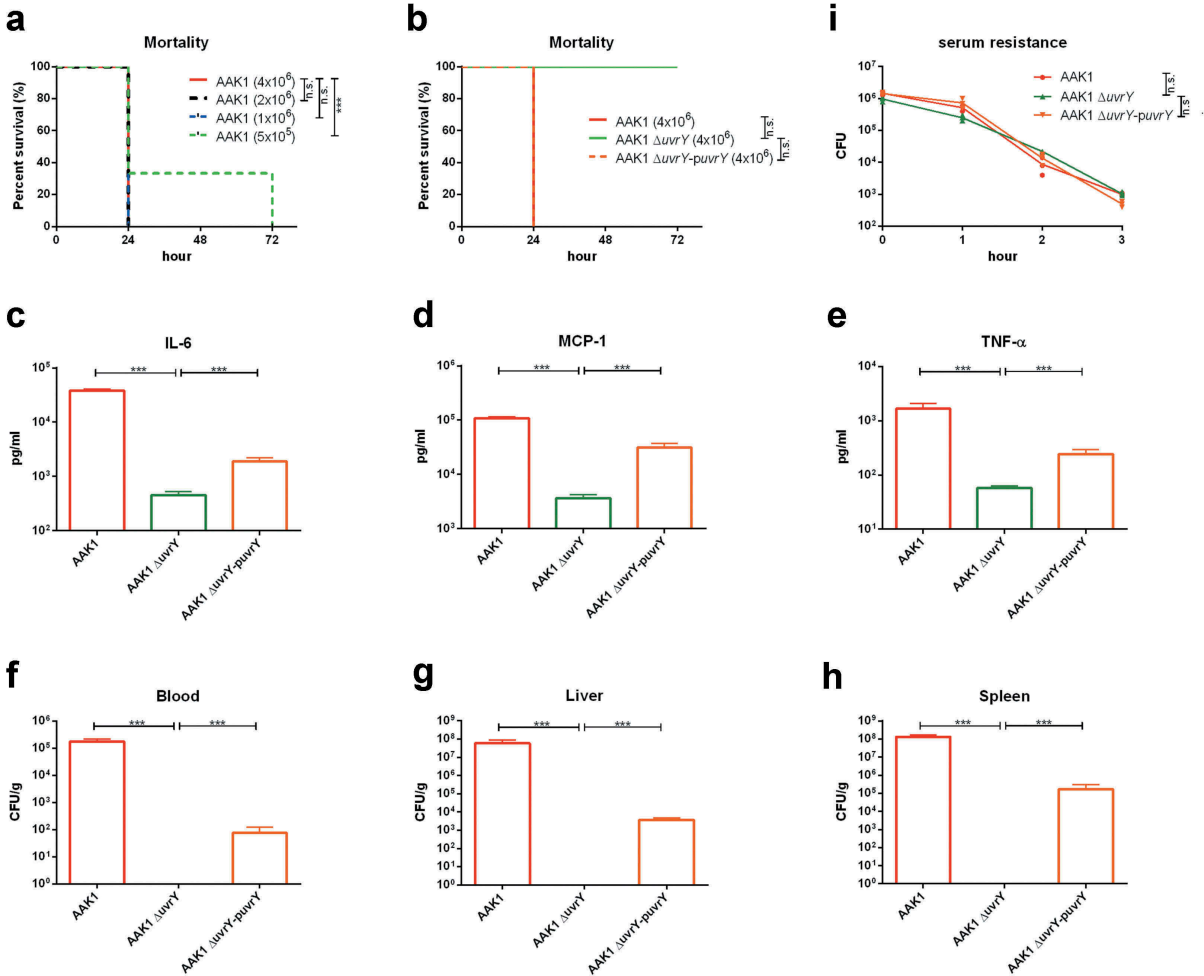
**UvrY is required for *A. dhakensis* virulence in a mouse infection model**. (a) Survival of mice infected with various doses of wild type *A.* *dhakensis* AAK1. (b) Survival of mice infected with the wild type *A.* *dhakensis* AAK1, uvrY mutant, and uvrY complement strains at high doses (4x10^6^ CFU). The levels of cytokine (c) IL-6, (d) MCP-1, and (e) TNF-α in mice infected with the wild type *A.* *dhakensis* AAK1, uvrY mutant and uvrY complement strains. Bacterial load in the (f) blood, (g) liver, and (h) spleen of mice infected with *A.* *dhakensis* AAK1, uvrY mutant, and the uvrY complement strain. (i) Differences in serum resistance among the *A.* *dhakensis* AAK1, uvrY mutant, and the uvrY complement strains were not significant. (***P < 0.001, **P < 0.01 and *P < 0.05).

To determine if the virulence attenuation of the *uvrY* mutant is cohesive in *C. elegans*, in cell, and in mammalian animals, we established an intraperitoneal (IP) infection of *A. dhakensis* in a mouse model. With the wild type *A. dhakensis* AAK1 strain, only a few mice survived for more than 24 hours after IP injection with a lower infection dose (5x10^5^ CFU) (Figure 4(a)). At a higher infection dose (4x10^6^ CFU) of the wide type strain, the mice were all dead within 24 hours. However, IP infection of the *uvrY* mutant at a dose of 4 × 10^6^ CFU resulted in no death of mice 72 hours post infection (Figure 4(b)). These results suggested that the virulence of *uvrY* mutant is attenuated in the murine model.

Inflammation is an important immune response against bacterial infection. The levels of inflammatory markers such as TNF-α, IL-6, and MCP-1, is all correlated with the severity of infection [31,46]. By analyzing the concentrations of IL-6 Figure 4(c), MCP-1 Figure 4(d) and TNF-α Figure 4(e) in mice serum, we found that the *uvrY* mutant did not cause severe inflammation, which occurred with the wild type AAK1 and the *uvrY* complement strain. We also measured the bacterial load in the blood Figure 4(f), liver Figure 4(g) and spleen Figure 4(h) of infected mice and the results showed the bacterial load of the *uvrY* mutant was significantly lower than the case for both the wild type AAK1 and *uvrY* complement strains in all organs. In the serum resistance assay, the wild type AAK1, *uvrY* mutant and *uvrY* complement strains all demonstrated serum resistance at similar levels (Figure 4(i)). These results suggested that the attenuation of the *uvrY* mutant is not related to alterations in the structure of the cell envelope. In other words, specific toxins rather than the components of the cell envelope are regulated by UvrY and required for its virulence in *A. dhakensis*.

**UvrY controls an array of virulence factors in *A. dhakensis***.

**Figure 5.**
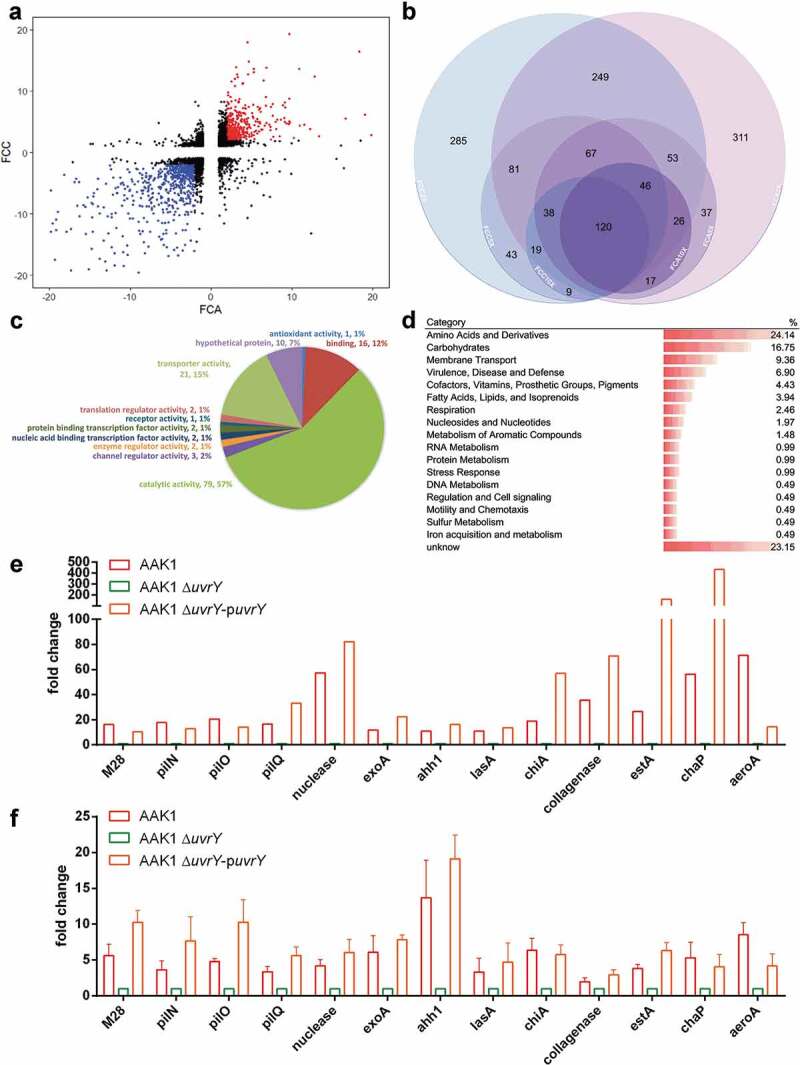
**Several virulence factors are regulated by UvrY in *A. dhakensis***. The transcriptome of UvrY was analyzed using an RNAseq analysis. (a) Scatter diagram of the fold change of in gene transcriptional levels in the *uvrY* mutant as compared to the wild type AAK1 (FCA) and in the *uvrY* mutant as compared to the *uvrY* complement (FCC), where each dot represents a transcript. The red dots are the transcripts with more than a two-fold increase in expression levels in the *uvrY* mutant as compared to the control strains. The blue dots are transcripts with more than a two-fold decrease in the expression levels in the *uvrY* mutant as compared to the control strains. (b) Venn diagram of the blue dots shown in Fig 5A. There are 120, 271, and 699 genes associated with the intersection of FCA10X and FCC10X, FCA5X and FCC5X, and FCA2X and FCC2X, respectively. (c) GO analysis of 120 candidate genes based on molecular function. (d) Category analysis of the 120 candidate genes using a RAST analysis. (e) Genes in the “Virulence, Disease and Defense” category shown in Fig 5D. (f) qRT-PCR of all genes belonging the “Virulence, Disease and Defense” category shown in Fig 5D.

The response regulator of a two-component system is usually a transcription factor [47]. To determine whether UvrY regulates the expression of virulence-associated molecules, the transcriptome of wild type strain and *uvrY* mutants were analyzed. The RNAseq results of *uvrY* mutant were compared to wild type AAK1 and *uvrY* complement strains. The genes with more than 2X-fold change were thought to be the potential candidates (Figure 5(a)). A total of 120 genes with expression at a lower transcription level in the *uvrY* mutant as compared to the wild type AAK1 and *uvrY* complement strains that achieved a 10X-fold difference were enrolled for analysis (Figure 5(b) and S6 File).

With Gene Ontology (GO) analysis, all 120 genes were sorted according to their already known function (Figure 5(c)). More than half of the genes exhibit catalytic activity. Expression of those genes involving in glycolysis and gluconeogenesis, however, is not significantly different between wild type and *uvrY* mutants. To clarify and highlight the potential route by which *uvrY* regulates virulence of *A. dhakensis*, the Rapid Annotation of microbial genomes using Subsystems Technology (RAST) [34,35] was also applied for further analysis. Among the 120 candidate genes, 6.9% of them were found in the Virulence, Disease and Defense subsystem of RAST (Figure 5(d)). According to the RAST analysis, the expression level of several well-known virulence factors in *Aeromonas* were in line with a change in the *uvrY* expression level, including pilus, protease, exo-toxin, collagenase, chitinase, aerolysin and hemolysin [9]. The RNAseq results of these RNAs (Figure 5(e)) were reconfirmed with qRT-PCR (Figure 5(f)) and showed a cohesive expression pattern where these RNAs all had lower expression levels in the *uvrY* mutant as compared to the wild type AAK1 or *uvrY* complement strains. These findings suggested that *uvrY* controls the expression of different virulence factors in *A. dhakensis*.

### Hemolysin ahh1, a type of pore-forming toxin, is regulated by *UvrY*

**Figure 6.**
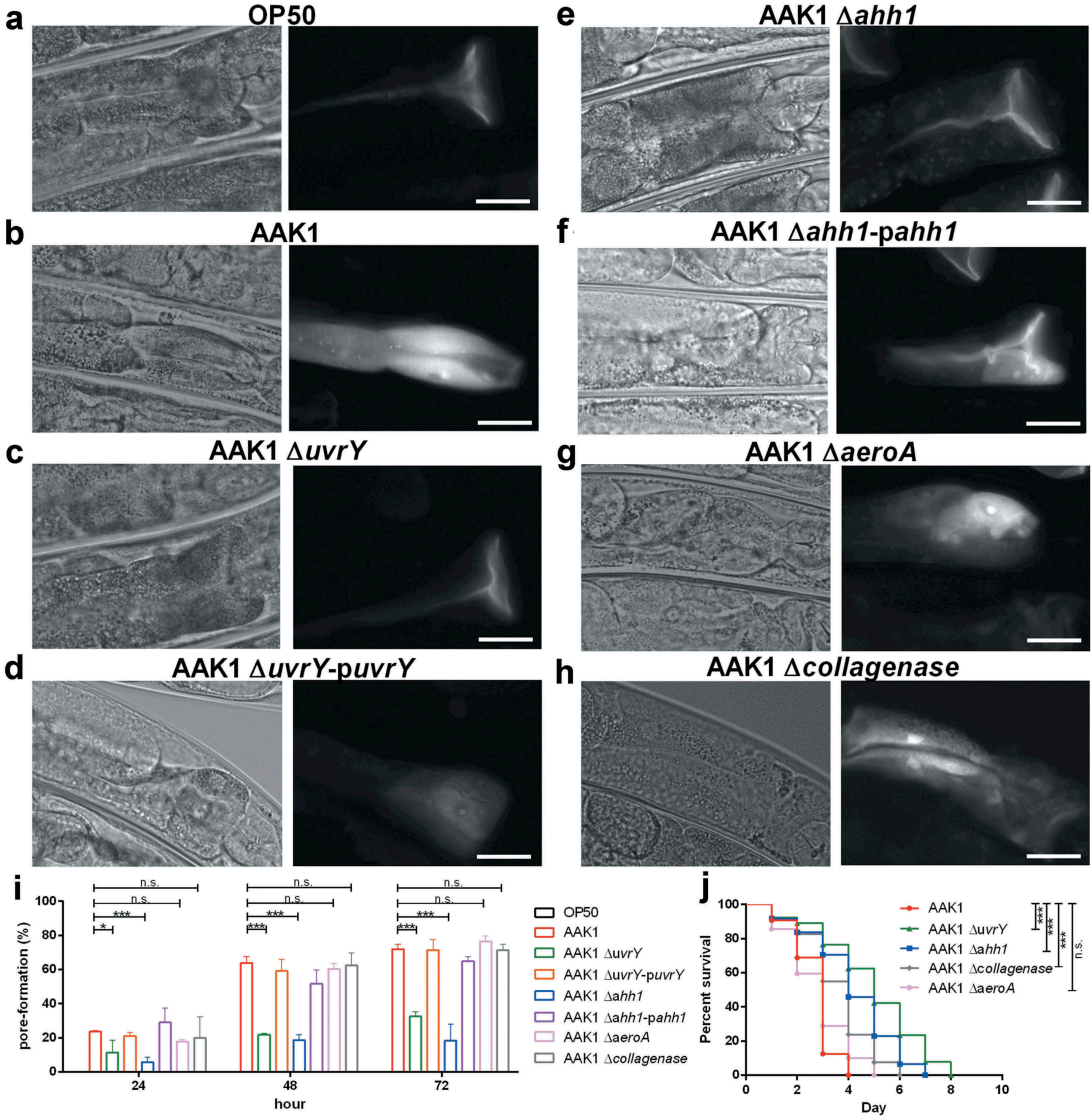
**Hemolysin (Ahh1), a pore-forming toxin is regulated by UvrY in *A. dhakensis***. The pore-forming phenotype in the intestine of *C. elegans* after infection with (a) *E. coli* OP50, (b) *A. dhakensis* AAK1, (c) the *A. dhakensis* AAK1 *uvrY* deletion mutant, (d) the *A. dhakensis* AAK1 *uvrY* complement, (e) the *A. dhakensis* AAK1 hemolysin *ahh1* deletion mutant, (f) the *A. dhakensis* AAK1 *ahh1* complement, (g) the *A. dhakensis* AAK1 aerolysin *aeroA* deletion mutant, and (h) the *A. dhakensis* AAK1 *collagenase* deletion mutant. The quantification of pore formation is summarized in (i). (j) Survival assay of *C. elegans* infected with *A. dhakensis* mutants. The scale bars in **(A)** to **(H)** are all 30 μm (***P < 0.001, **P < 0.01 and *P < 0.05).

Clinically, a severe bacterial infection, such as soft tissue necrotizing fasciitis, is usually caused by pore-forming toxins, including hemolysin and aerolysin in *Aeromonas*, cytolysin in *Vibrio* and leucocidin in *Staphylococcus aureus* [48]. To determine if the attenuation of the *uvrY* mutant contributes to the reduced production of pore-forming toxins, a pore-forming assay with propidium iodide (PI) was conducted using the *C. elegans* infection model [36,37]. Propidium iodide (PI), a dye with red fluorescence cannot pass through the cell membrane unless the membrane integrity is broken. The intestinal cells of *C. elegans* were intact when they were fed with normal food source *E. coli* OP50 Figure 6(a-i). The retention of PI dye in the gut indicated the absence of pore-formation in the intestine cells. Comparably, the dye was dispersed into the intestinal cells of the nematodes after infection with *A. dhakensis* Figure 6(b-i). In line with our prediction, worms fed with the *uvrY* mutant did not show the pore-formation phenotype Figure 6(c-i), and by contrast, the worms infected with the *uvrY* complement strain were similar to the wild type *A. dhakensis* AAK1 Figure 6(d-i).

To clarify whether the *uvrY*-regulated pore-forming toxins contribute to the virulence of *A. dhakensis*, isogenic mutants of hemolysin (*ahh1*), aerolysin (*aeroA*), and *collagenase* were generated. Interestingly, only worms infected with the *ahh1* mutant failed to demonstrate pore-forming phenotypes, as was also the case for the worms infected with OP50 and the *uvrY* mutant Figure 6(e-i). The worms fed with the *aeroA* mutant and *collagenase* mutant exhibited obvious pore-formation phenotypes Figure 6(g-i). The *ahh1* complement strain rescued the pore-formation phenotype in *C. elegans* Figure 6(f-i).

To link pore-formation pathology and virulence, the standard *C. elegans* survival assay was conducted (Figure 6(j)). The *ahh1* as well as the *uvrY* mutants were less virulent as compared to with the a*eroA* and *collagenase* mutants. Although the *aeroA* and *collagenase* mutants did show pore-formation phenotypes in *C. elegans*, these two virulence factors still exhibited toxicity to a lesser degree in *C. elegans*. In summary, hemolysin *ahh1*, being one of the most potent virulence factors in *A. dhakensis*, is regulated by *uvrY* and has the characteristics of a pore-forming toxin.

**UvrY is required for the expression of hemolysin Ahh1**.

**Figure 7.**
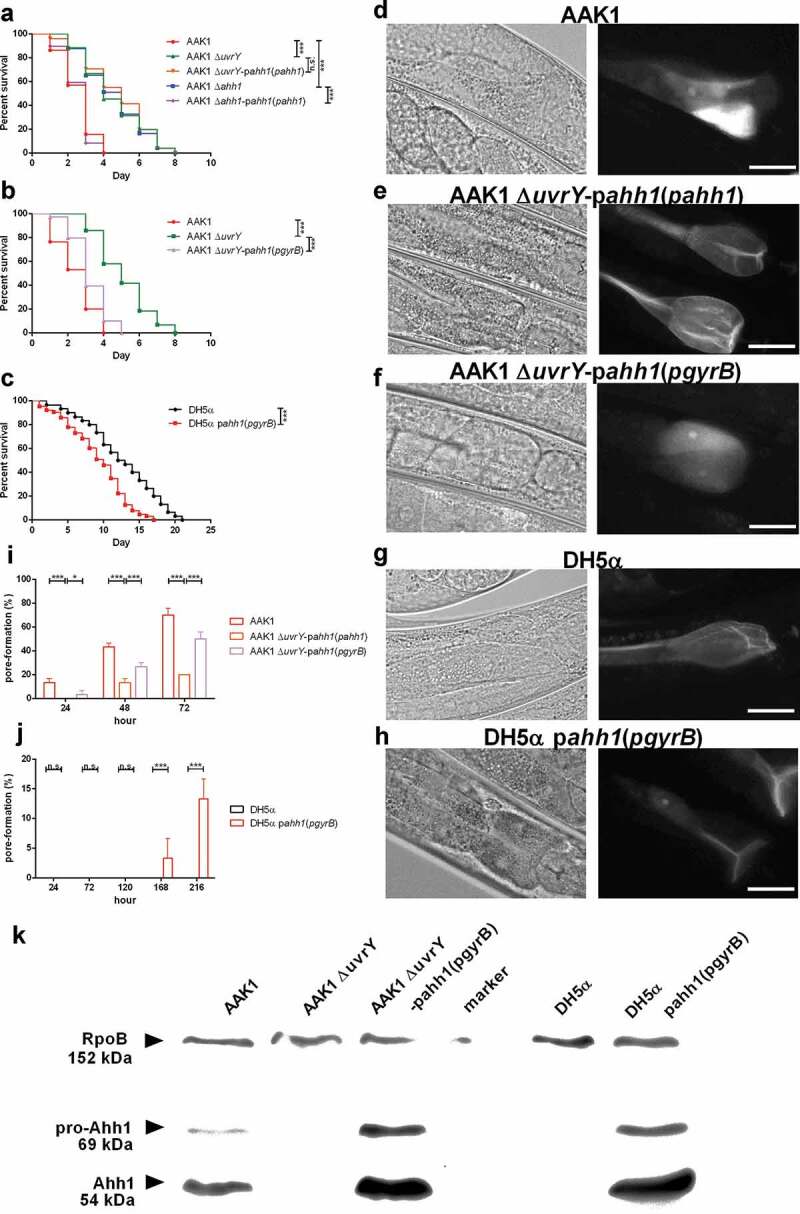
**UvrY is required for the expression of hemolysin Ahh1 in *A. dhakensis***. (a) Survival assay of *C. elegans* infected with *A. dhakensis* mutants. Complementation of *ahh1*-promoter-driven *ahh1* did not rescue the toxicity of the *uvrY* mutant. (b) *A. dhakensis* mutants with complementation of *gyrB*-promoter-driven *ahh1* recovered the toxicity in the *C. elegans* survival assay. (c) *C. elegans* infected with Ahh1 over-expressed bacteria had lower survival rates. The pore-formation phenotype in the intestine of *C. elegans* infected with (d) *A. dhakensis* AAK1 (e) the *ahh1*-promoter-driven *ahh1* complement to *uvrY* deletion mutant (f) the *gyrB*-promoter-driven *ahh1* complement to *uvrY* deletion mutant (g) *E. coli* DH5α and (h) Ahh1 over-expressed *E. coli* DH5α. The quantification of pore-formation is summarized in (i) and (j). (k) Western blot of Ahh1 in the cell lysates of different strains. The scale bars in **(D)** to **(H)** are all 30 μm. (***P < 0.001, **P < 0.01 and *P < 0.05).

To determine whether UvrY is necessary for the expression of Ahh1, the Ahh1 complement strain under a *uvrY* deletion background was generated. Interestingly, the virulence of the *ahh1*-promoter-driven Ahh1 complement strain under the *uvrY* deletion background [AAK1 Δ*uvrY*-p*ahh1*(*pahh1*)] was not reversed. In contrast, the virulence of the Ahh1 complement strain under the *ahh1* deletion background [AAK1 Δ*ahh1*-p*ahh1*(*pahh1*)] was reversed in the *C. elegans* model (Figure 7(a)). The toxicity of the Δ*uvrY*-p*ahh1*(*pahh1*) strain was reduced, similar to the *uvrY* deletion mutant and the *ahh1* deletion mutant. However, the strain with complementation of a constitutively active house-keeping gene *gyrB* promotor-driven Ahh1 strain in the *uvrY* deletion mutant [AAK1 Δ*uvrY*-p*ahh1*(*pgyrB*)] increased the toxicity Figure 7(b). Over-expression of Ahh1 in a nonpathogenic *E. coli* DH5α showed an increase in toxicity Figure 7(c). Similar to AAK1 Figure 7(d-I), a higher proportion of worms infected with AAK1 Δ*ahh1*-p*ahh1*(*pahh1*) strain showed a pore-formation phenotype (Figure 7(f-i)), in contrast to a lower proportion in the AAK1 Δ*uvrY*-p*ahh1*(*pahh1*) strain (Figure 7(e-7i)). The pore-formation rate was also increased in the DH5αp*ahh1*(*pgyrB*) strain as compared to DH5α (Figure 7g, 7h and 7j). The pore-formation ratio was correlated to the virulence in terms of inverse survival in *C. elegans*.

To reconfirm the increase in toxicity was due to an effective Ahh1 complementation, a western blot analysis was performed on Ahh1. With a polyclonal anti-Ahh1 antibody, Ahh1 proteins were detected only in the wild type AAK1, AAK1 Δ*uvrY*-p*ahh1*(*pgyrB*), and DH5αp*ahh1*(*pgyrB*) (Figure 7k). It is thus suggested that the toxicity of bacteria results from the expression of Ahh1. Taken together, UvrY controls the virulence of hemolysin Ahh1 and is required for the full expression of virulence in *A. dhakensis*.

## Discussion

**Figure 8.**
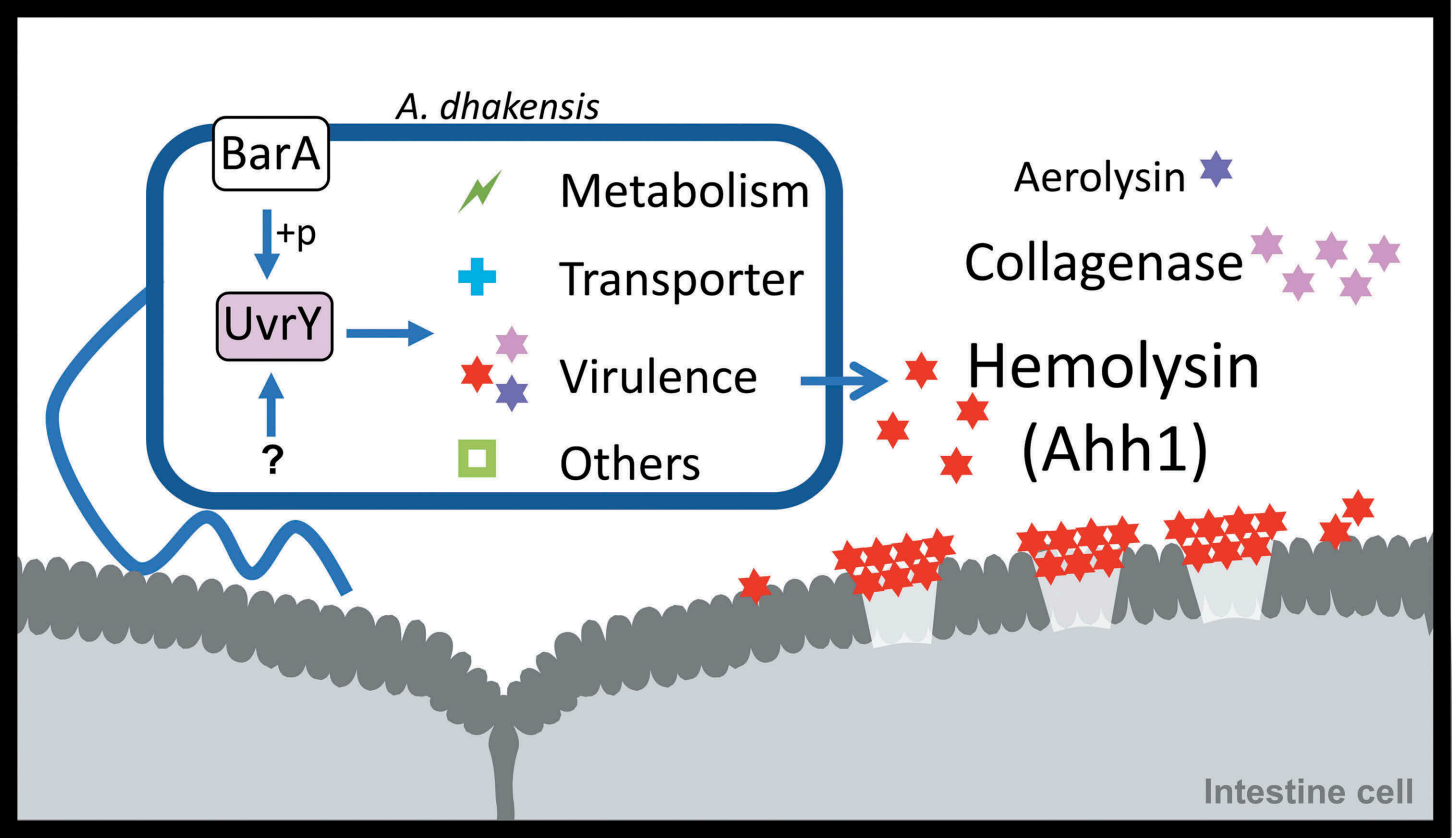
**Schematic diagram of UvrY-mediated pathogenesis in *Aeromonas dhakensis***. Activation of UvrY is induced by BarA or other unknown molecules. UvrY regulates the expression of Ahh1, the most potent toxin as well as other virulence factors in *A. dhakensis*. Ahh1 results in pore-formation in cells and cause severe infection in hosts.

In this research, we generated a transposon mutation library of *A. dhakensis* and surveyed the *A. dhakensis* mutants with attenuated virulence in *C. elegans*. We identified UvrY and discovered that UvrY regulates the expression of hemolysin Ahh1 as well as other virulence factors in *A. dhakensis*. The pore-formation caused by Ahh1 is a vital pathogenesis of *A. dhakensis* infection in *C. elegans* (Figure 8).

In our mutant library, mutation of genes involved in flagella biosynthesis are abundant. These hits present reduced virulence in *C. elegans* and suggest that flagella biosynthesis is also important for the virulence of *A. dhakensis*. Flagella is a key mobility structure responsible for chemotaxis and biofilm formation [49,50]. Mutation of flagella may cause a decrease in attachment to the cell surface and reduce the infection efficiency in hosts.

We identified UvrY as a key component in the control of the full virulence of *A. dhakensis*. The similarity of the UvrY homologue among different bacteria genera is shown in S7A Fig. Using a RAST analysis, the UvrY of *A. dhakensis* AAK1 and its homologue in other bacteria genera were demonstrated, as shown in S7B Fig and S7 C Fig. It is believed that the homologue of UvrY plays a similar role in regulating virulence, such as in *E. coli, Salmonella, Pseudomonas*, and *Vibrio* [43]. Since UvrY or UvrY -like regulators control a broad range of virulence factors in pathogenic bacteria, further studies are warranted to figure out the exact mechanism mediated by UvrY.

As a member of a two-component system, the upstream sensor kinase which phosphorylates UvrY is also thought to play a role in determining the virulence of *A. dhakensis*. The most well-known sensor kinase of UvrY is BarA. Studies have found that BarA phosphorylates UvrY D54 residue [11,42,43]. In this study, however, complementation of UvrY D54E (constitutively active form) and D54A (inactive form) both rescued the virulence (Figure 3(e,f)). It suggests that the phosphorylation of D54 residue is not essential for UvrY-associated virulence. Possible interpretations for this finding include (1) D54 of UvrY is phosphorylated by BarA, however, is not essential for UvrY-associated toxicity; (2) Beside BarA, there is another phosphate donor that phosphorylates UvrY at non-D54 residue.

*A. dhakensis barA* mutant is as attenuated as *uvrY* mutant (Figure 3(a,b)), However, the increased toxicity of Δ*barA-*p*uvrY* in (Figure 3(c,d)) suggested that without the phosphorylation by BarA, overexpressed UvrY is able to activate the downstream signaling. This result is similar to previous finding that UvrY may be phosphorylated by other molecule, such as acetyl-phosphate [44,45]. Interestingly, *barA* mutant which showed attenuated virulence still maintained a copy of *uvrY* gene. These evidences suggest that the attenuated virulence of *barA* mutant may come from suppressed expression of downstream signaling which is regulated by different mechanism. Base on this, we tested the expression of Ahh1 in *barA* mutant (S8 Fig). The result showed that *barA* mutant still produced pro-Ahh1, a pro-form of Ahh1. In contrast, expression of pro-Ahh1 and Ahh1 is largely deficient in *uvrY* mutant (Figure 7k). In a brief summary, the roles of BarA, UvrY, BarA-UvrY two-component system, are complex then as expected. Further studies for identification of other factors which play a role in regulating virulence are needed.

Based on the transcriptome results, UvrY is believed to control the expression of several virulent genes. However, how can UvrY regulate these downstream virulence genes is still unclear. As a response regulator in a two-component system, UvrY is thought to be a transcription factor. It is possible that UvrY participates in the transcription of these virulence directly. However, the possibility also exists that UvrY affects the expression of some downstream virulent genes indirectly. For example, UvrY transcribes small RNA *csrB, csrC* [14,15], *rsmY*, and *rsmZ* [51–53], and these small RNAs bind to translation initiator CsrA to suppress the function of CsrA, which is an RNA binding protein influencing post-transcriptional regulation and causing changes in translation [54]. The consensus binding sequences of UvrY at the promoter region of small RNAs is not clearly understood [11,53,55]. In addition, the sequences of these potential binding regions are not cohesive. The lack of consistent binding sequences increases the possibility that UvrY regulates a broad range of RNAs that subsequently activate the expression of downstream virulent genes.

In our case, UvrY was required for the expression of hemolysin *ahh1*. We therefore provide some proposals which explain how UvrY regulate *ahh1* (1) UvrY binds to the *ahh1* promoter and transcribes *ahh1* directly. (2) CsrA, the downstream of UvrY, affects the expression of *ahh*. (3) Other unknown components, which are regulated by UvrY, bind to either the promoter of *ahh1* or the mRNA of *ahh1*. We screened transcription factors which were affected by UvrY, and identified NarL and PdhR as potential transcription regulators of *ahh1* based on RNAseq analysis. Since NarL and PdhR have been suggested to be negative transcription regulators in previous studies [56,57], the transcription levels of *narL* and *pdhR* are increased as expected in the RNAseq profiles of *uvrY* mutant (S6 file), and are supposed to repress *ahh1*expression consequently. However, further research to prove this hypothesis is warranted.

Although UvrY regulates many virulence factors, the complementation of Ahh1 to the *uvrY* mutant rescues full virulence. In contrast, the well-known aerolysin, an important pore-forming toxin of *Aeromonas* in the cell and mouse models [58,59], is not associated with *A. dhakensis* virulence in *C. elegans*. This phenomenon may be explained by the fact that aerolysin is not an effective pore-forming toxin to *C. elegans*. Similarly, *collagenase* may contribute to the limited virulence of *A. dhakensis* toxicity in *C. elegans*. As has been previously found [60], it is possible that *collagenase* is not the central virulence factor of *A. dhakensis*. Due to the virulent traits of Ahh1, further study to the determine its pathogenesis is necessary.

Because UvrY is prevalent in a variety of bacteria genera and acts as a key regulator of many virulence factors, UvrY is a potential therapeutic target of antimicrobial agents. Previous studies have shown that two-component signaling systems are potential drug targets when developing new antimicrobial agents [61]. Therapy with novel agents inhibiting *uvrY* expression along with traditional antibiotics may lead to additional benefits for patients suffering from severe sepsis.

## Supplementary Material

Supplemental Material
