# Supplementary material for: UvrY is required for the full virulence of Aeromonas dhakensis
Source: Virulence. 2020 May 20;11(1):502–20. doi: 10.1080/21505594.2020.1768339 (PMC7250320; doi:10.1080/21505594.2020.1768339)
Supplement: Supplemental Material [file kvir-11-01-1768339-s001.zip › Supporting information.docx]

**Supporting information**

**S1 Fig. GO analysis of the 31 candidate genes responsible for virulence attenuation in *Aeromonas dhakensis*.** GO analysis of hits based on **(A)** biological process, **(B)** cellular component, and **(C)** protein class.

**S2 Fig. The summary of mobility, auxotroph, and biofilm results of the 31 *A. dhakensis* mutants with attenuated virulence. (A)** swimming, **(B)** swarming, **(C)** auxotroph, and **(D)** biofilm formation. *A. dhakensis* AAK1, *S. aureus* ATCC 29213, *B. subtilis* 3610, and *E. coli* OP50 are the controls in the corresponding chart.

**S3 Fig. Summary of the growth curves of the 31 *A. dhakensis* mutants with attenuated virulence.** The continuous growth of bacteria was measured using the OD-Monitor C&T (Taitec, Japan). Transposon mutants were grown in 5 ml of LB broth and incubated at 37 ^o^C in the OD-Monitor. The OD600 values of each bacteria were obtained every 30 minutes.

**S4 Fig. Summary of the virulence of mutants in terms of survival of *C. elegans*.** Approximately 50 L4 worms were transferred to an NG plate with each transposon mutants separately. Live worms were counted and transferred daily. All survival assays were performed at 20 ^o^C.

**S5 Fig. Diagrams of UvrY mutants. (A)** The insertion site of the mini-Tn10 transposon of AA078G03. **(B)** Replacement of a kanamycin resistant cassette (*nptII*) in the *uvrY* deletion mutant using homologues recombination.

**S6 File. Partial results of RNAseq among wild type AAK1, *uvrY* mutant, and *uvrY* complement strains.** The file displays the annotations of 699 transcripts with a greater than two-fold decrease of RNAseq results in the *uvrY* mutant compared to the control strains.

**S7 Fig. Homologue and alignment of UvrY in different bacteria. (A)** The identity (%) of the UvrY homologue in different bacteria. **(B)** Diagram from RAST of the UvrY homologue in different bacteria genera. **(C)** Sequence alignment of UvrY in different bacteria genera.

**S8 Fig. The expression of Ahh1 in *barA* mutant.** Western blot of Ahh1 in the cell lysates of *A. dhakensis* AAK1 and *barA* mutant.
