# Supplementary figures and images for: UvrY is required for the full virulence of Aeromonas dhakensis
Source: Virulence. 2020 May 20;11(1):502–20. doi: 10.1080/21505594.2020.1768339 (PMC7250320; doi:10.1080/21505594.2020.1768339)

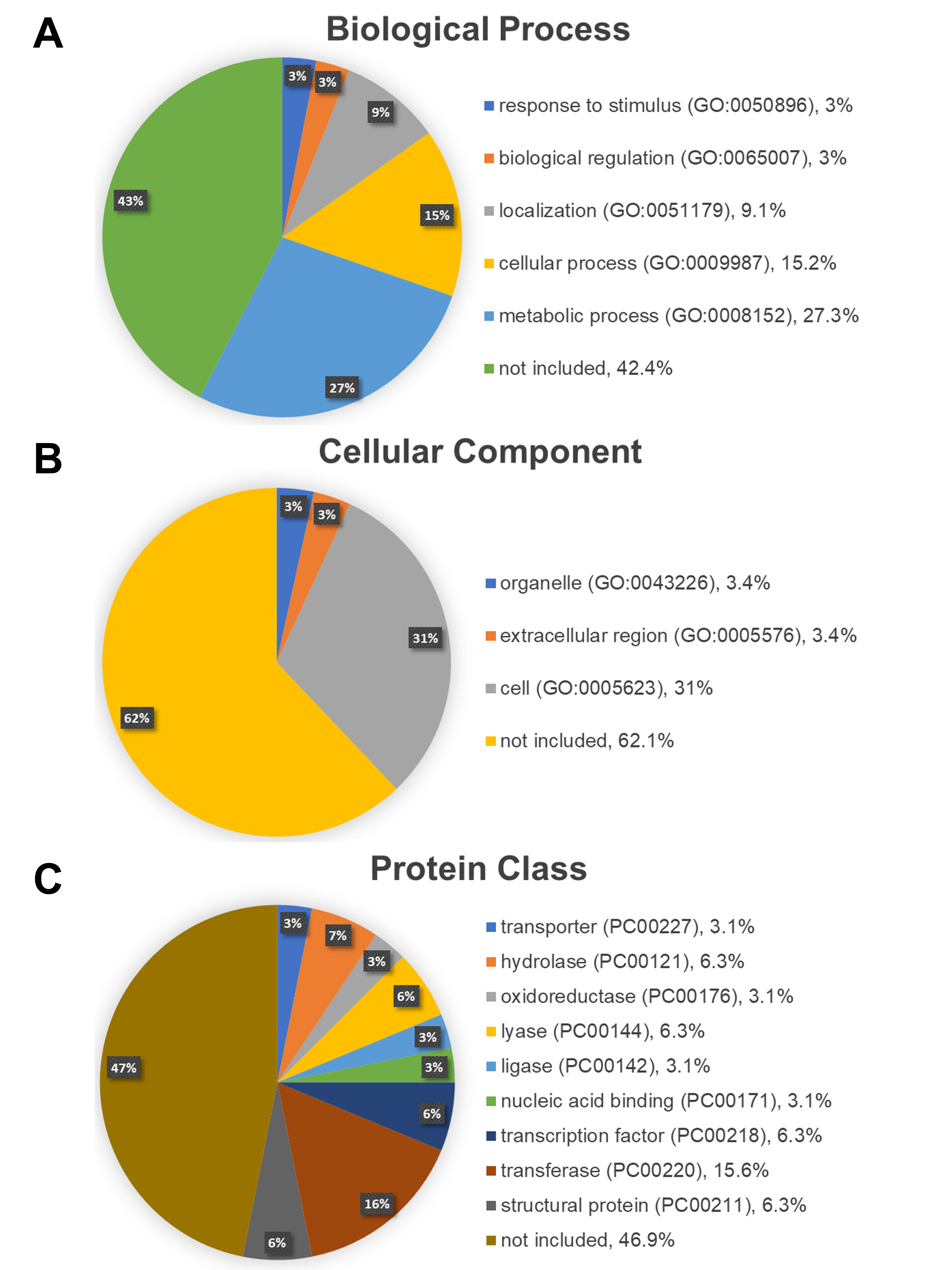

Supplement: Supplemental Material [file kvir-11-01-1768339-s001.zip › s1N.tif]

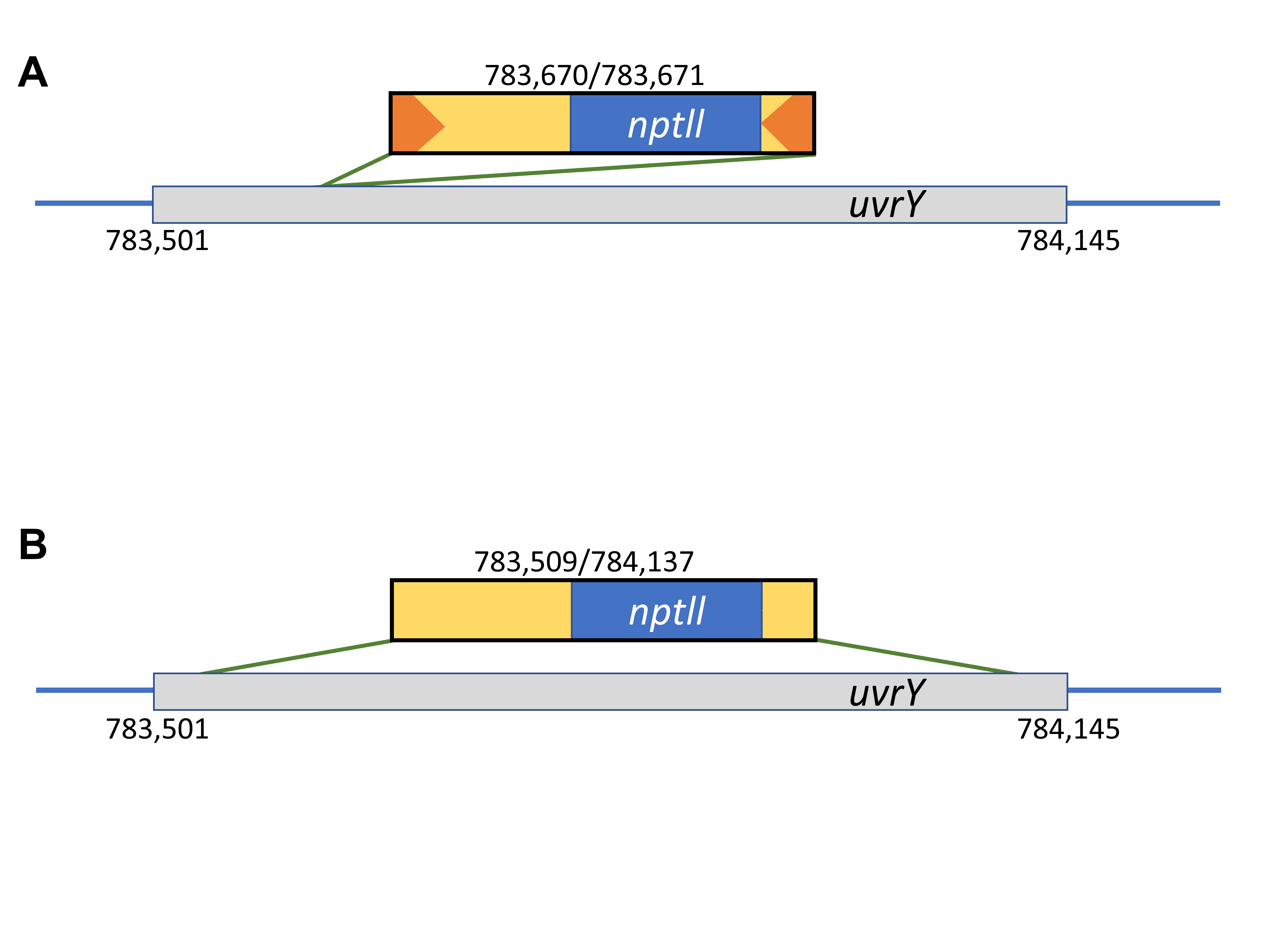

Supplement: Supplemental Material [file kvir-11-01-1768339-s001.zip › S5.tif]

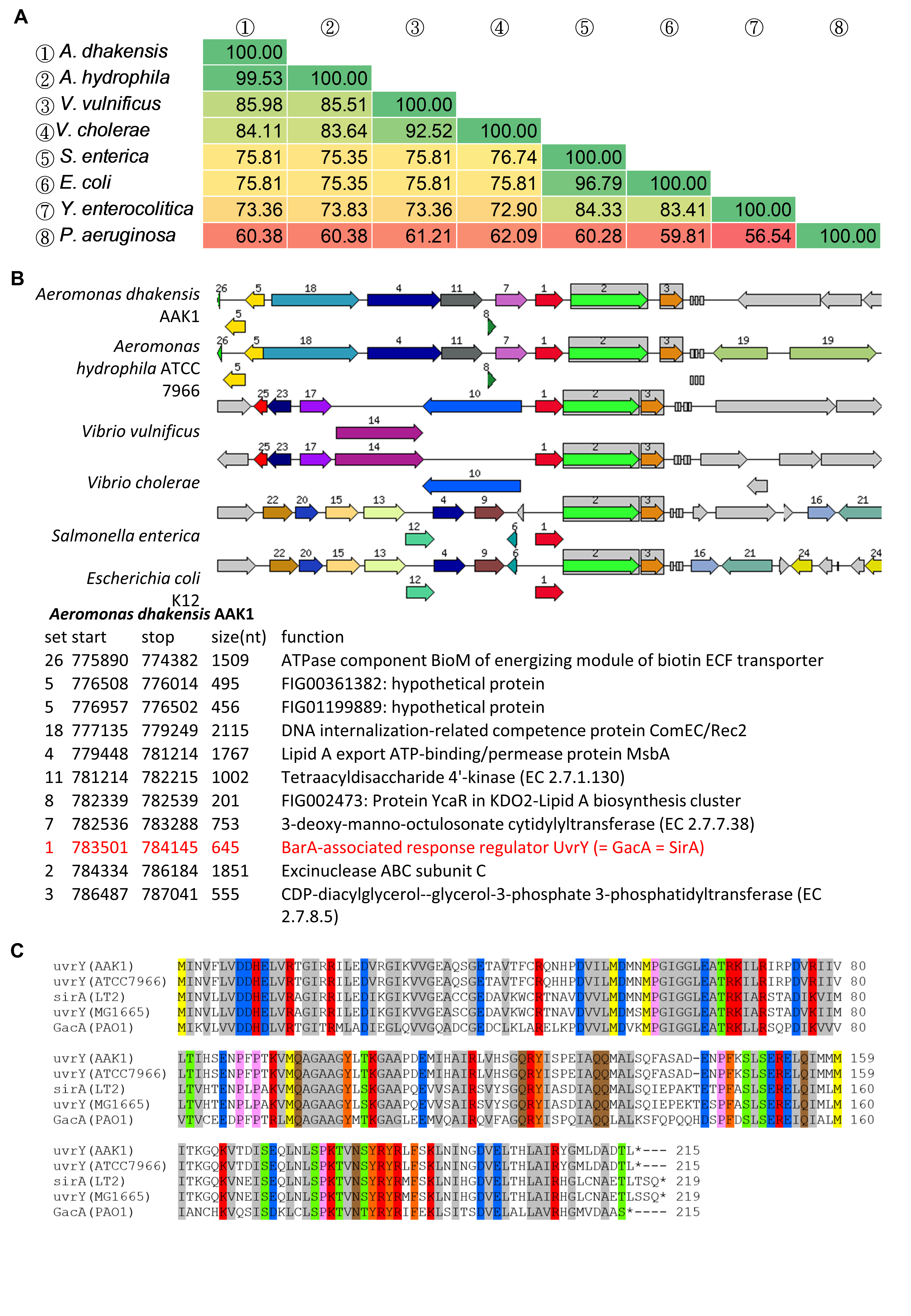

Supplement: Supplemental Material [file kvir-11-01-1768339-s001.zip › S7.tif]
